# Rac1 regulates skin tumors by regulation of keratin 17 through recruitment and interaction with CD11b+Gr1+ cells

**DOI:** 10.18632/oncotarget.2030

**Published:** 2014-05-28

**Authors:** Rongyi Chen, Meng Fu, Guoxue Zhang, Ying Zhou, Shaojun Zhu, Juanjuan Liu, Dong Wang, Anmei Deng, Zhipeng Wang

**Affiliations:** ^1^ Department of Dermatology, Affiliated Hospital of Guangdong Medical College, Zhanjiang, China; ^2^ Department of Dermatology, Xijing Hospital, Fourth Military Medical University, Xi'an, China; ^3^ The Helmholtz Sino-German Research Laboratory for Cancer, Department of Pathology, Tangdu Hospital, the Fourth Military Medical University, Xi'an, China; ^4^ Key Laboratory of Gastrointestinal Pharmacology of Chinese Materia Medica of the State Administration of Traditional Chinese Medicine, Department of Pharmacology, School of Pharmacy, Fourth Military Medical University, Xi'an, China; ^5^ Department of Laboratory Diagnostic, Changhai Hospital, Second Military Medical University, Shanghai, China

**Keywords:** Rac1, keratin17, Skin tumor

## Abstract

Rac1 is a member of the Rho family of small GTPases that control cells proliferation, differentiation, migration, and inflammation. Rac1 is crucial in tumorigenesis and development. Keratin17 and CD11b+Gr1+ cells are considered to regulate skin inflmmation. Here we discuss the regulation of Rac1 on skin tumor formation and its relationship. In samples from human skin squamous cell carcinoma (SCC), Rac1 activity was higher in cancer tissues than in normal skin and activity correlated with keratin 17 overexpression. In a DMBA/TPA-induced mouse skin tumor model, inhibition of Rac1 activity and depletion of CD11b+Gr1+ cells resulted in significant tumor formation. TPA induced recruitment of CD11b+Gr1+ cells into dermis; however, Rac1 inhibitor abolished this recruitment. *In vitro*, Rac1 induced interferon (IFN) and interlukin (IL6) production in keratinocytes, repression of keratin 17 inhibited IFN and IL6 production induced by Rac1. Moreover, both inhibition of Rac1 activity and repression of keratin 17 restricted proliferation and induction of differentiation in keratinocytes. Coculture of CD11b+Gr1+ cells with keratinocytes activated Wnt pathway in keratinocytes, resulting in enhanced Rac1 activity, overexpression of keratin 17, and hyperproliferation of keratinocytes. Our results suggested that hyperactive Rac1 recruited and interacted with CD11b+Gr1+ cells, inducing keratin 17-regulated inflammation and promoting skin tumor formation.

## INTRODUCTION

Squamous cell carcinoma (SCC) is the second most common type of skin cancer [1]. Risk factors for SCC include ultraviolet radiation from sun exposure and sunbeds, and skin injury such as burns, scars, long-standing sores, X-rays or exposure to chemicals such as arsenic and petroleum byproducts. Recently, chronic skin inflammation was identified as a factor in the development of SCC [2,3]. Chemically induced skin carcinogenesis models rely on inflammatory agents such as 12-O-tetradecanoylphorbol 13-acetate (TPA) that create inflammatory microenvironments that promote tumor formation and progression [4].

Tumor initiation and progression can require complex interactions between cells and the immune system [5]. Intrinsic inflammation pathways in tumor cells as well as extrinsic pathways that interact with tumor-infiltrating leukocytes are known to contribute to tumor progression. Tumor-infiltrating immune cells either antagonize or promote cancer development, depending on the cell type. For instance, macrophages and neutrophils [6] promote tumor growth by a variety of mechanisms. CD11b+ Gr1+ myeloid cells, a myeloid macrophage-dendritic cell lineage, are known to be myeloid-derived suppressor cells (MDSCs) [7]. CD11b+ Gr1+cells are significantly increased in tumors that are associated with impaired immune reactivity [8,9]. CD11b+ Gr1+cells were found overproduced in the bone marrow and spleens of tumor-bearing mice, as well as in the peripheral blood of cancer patients [10, 11]. Furthermore, CD11b+ Gr1+cells contribute directly to tumor development and vascularization by producing matrix metalloprotease 9 and differentiating into endothelial cells[12]. Despite data defining the tumor-promoting function of CD11b+Gr1+ cells, their interaction with keratinocytes and involvement in skin tumor formation is unclear.

Keratin 17, a myoepithelial keratin, is expressed under various pathological conditions, including psoriasis and cutaneous allergic reactions, and is not found in healthy epidermis. Hence, keratin 17 is considered to be a hallmark of psoriasis, a skin inflammatory disease with keratinocyte hyperproliferation [13]. Interferon (IFN)-gamma has long been recognized for its crucial role in inflammation and tumor formation [14]. IFN induced keratin 17 overexpression is part of a crucial autoimmune loop in psoriasis development. Keratin 17 expression correlates with tumor progression and poor prognosis in many cancers such as gastric adenocarcinoma and ovarian cancer [15]. However, the functions of keratin 17 in SCC is poorly understood.

Another factor involved in both skin and immunity is *RAC1*, which is proposed to regulate crosstalk between the epidermis and immune cells [4]. Rac1 is a member of the GTPase family that is activated by binding to GTP to regulate reorganization of the cytoskeleton, motility, proliferation, apoptosis, and differentiation [16]. We previously found that Rac1 is responsible for DMBA/TPA-induced skin tumor formation using keratinocytes restricted Rac1 knockout mice. Activated Rac1 promotes Erk-dependent hyperproliferation by Pak1-mediated Mek activation. Rac1 was also required for Pak2-dependent hyperactivation of Akt [17]. Upregulation of Rac1 activity by Wnt3a temporally correlates with enhanced p120-catenin binding to Rac1 and Vav2. The role of Rac1 in inflammation driving SCC initiation and development is unknown. Therefore, in this study, we have investigated the role of Rac1-mediated inflammation in skin tumorigenesis, specifically, its association with keratin 17 and CD11b+Gr1+ cells.

## RESULTS

### Rac1 hyperactivation in squamous cell carcinoma correlates with keratin 17 overexpression

**Figure 1:**
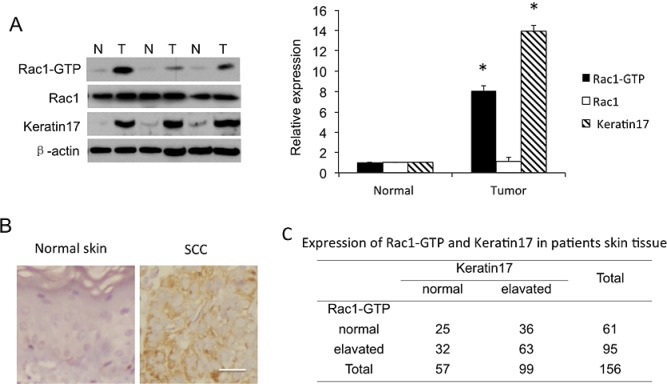
Rac1 activity as Rac1-GTP and Rac1 and keratin 17 expression in human skin squamous cell carcinoma (SCC) tissue A, Western blot of SCC patient tissues. Shown are representatives of equal amounts of cell lysate from SCC tissues (T) and paired normal tissue (N) from patients. Western blotting density was quantified from 156 samples. * p<0.01 compare to normal tissue. B, Immunohistochemistry for keratin17. Normal, adjacent normal tissue; cancer, SCC tissue. Scale bar: 50 μm. C, Expression of Rac1-GTP and keratin 17 in 156 patient skin tissue samples.

To elucidate Rac1 function in SCC development, we examined Rac1 expression and activity as Rac1-GTP, as well as keratin 17 expression in specimens from 156 SCC patients. Rac1-GTP levels significantly increased in the tumor tissues of 87 (56%) of the 156 SCC patients compared to adjacent normal tissue, suggesting hyperactivation of Rac1 in tumor tissue. Representative examples are in Figure 1A, B. Keratin 17 expression was elevated in 62% (98 of 156) of carcinoma samples. In addition, in 78% (63 of 95) Rac1-hyperactivated tissue samples, keratin 17 was overexpressed (Figure 1C). Thus, Rac1-GTP showed a positive correlation with keratin 17 expression. These results suggested that both Rac1 and keratin 17 were important in SCC tumorigenesis, and hyperactivated Rac1 might be related to keratin 17 overexpression.

### Rac1 activity affects skin tumor formation through regulation of cell proliferation and differentiation

**Figure 2:**
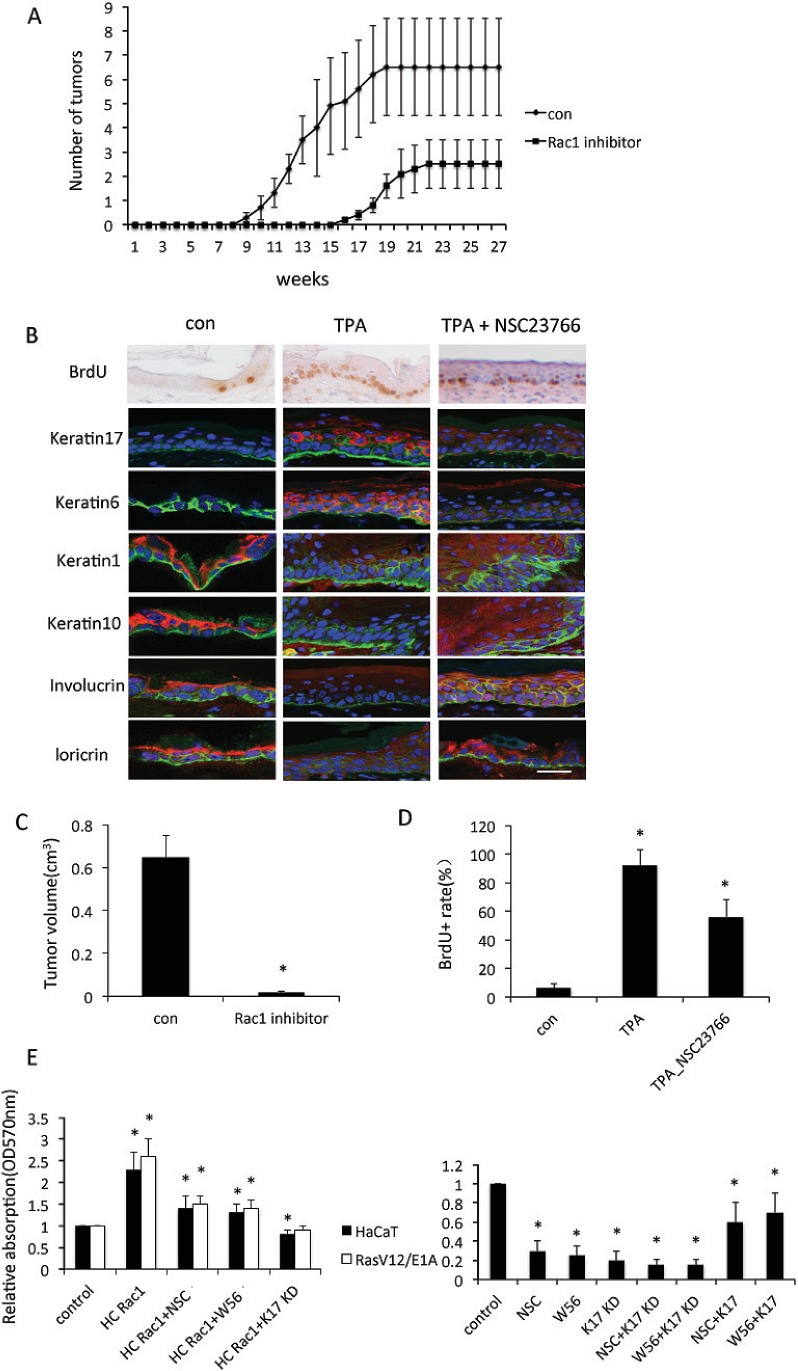
Rac1 is involved in keratinocyte proliferation A, 8 week old mice were subjected to DMBA/TPA-induced skin carcinogenesis for 30 weeks. Mice were separated with two groups (n = 10): Control (con) mice treated with DMBA/TPA only; mice (Rac1 inhibitor) were treated with 10 mM Rac1 inhibitor NSC23766 30 min prior to TPA treatment. Average number of tumors per mouse. B, Staining for BrdU incorporation, keratin 6, keratin 1, keratin10, keratin17, involucrin or loricrin (red). α6 (green) was used to stain basal layer. DAPI (blue) counterstaining indicates nuclei. Scale bar: 20 μm. Nontreated mice (con), TPA-treated mice (TPA), or TPA+NSC23766 treated mice (TPA+NSC23766). C, Quantification of tumor volumes at the endpoint of DMBA/TPA treatments. * p<0.01 compare to NSC23766 untreated mice. D, Quatification of BrdU positive cells in epidermis. * p<0.01 compare to control. E, Proliferation of keratinocytes. Keratinocyte cell line HaCaT or RasV12/E1A-transfected keratinocytes were transfected with high-cycling (HC) Rac1 plasmid or small hairpin RNA against keratin 17 (KD), or treated with Rac1 inhibitor (NSC or W56). Proliferation of keratinocytes was measured by crystal violet assay. Assays were performed in triplicate. The means with SD are shown (n=8 replicates/group).

Based on our findings that Rac1 was hyperactivated in SCC tissue, we used a mouse skin tumor model induced by DMBA/TPA to test the function of Rac1 in SCC carcinogenesis. In control mice, the first papillomas were observed 9 weeks after the DMBA treatment. After 19 weeks, all 10 control mice developed tumors (Figure 2A) with an average number of about 7 tumors per mouse. Littermates were treated with the Rac1 inhibitor NSC23766 painted onto the mice. This resulted in first tumors being observed at 16th week after DMBA treatment with an average of three tumors per mice with all mice again developing tumors. Tumor volumes were also significantly smaller in NSC23766 mice, compared to control mice(Figure2C). These data indicated that Rac1 function in keratinocytes was important for the formation of skin tumors.

To investigate the mechanism by which Rac1 promoted tumors, we analyzed proliferation and differentiation in skin cells from the backs of DMBA/TPA-treated and untreated mice. DMBA/TPA-induced hyperproliferation was determined by BrdU incorporation. Figure 2B, D shows that inhibition of Rac1 activity markedly reduced the number of proliferative cells. These results suggested that Rac1 was essential for DMBA/TPA-induced keratinocyte hyperproliferation *in vivo*. Moreover, DMBA/TPA induced an increase in keratin 17 and keratin 6 expression, which is a important marker for epidermis proliferation in controls and this increase in expression was lower in mice in which Rac1 activity was inhibited.

*In vitro*, we cultured immortalized HaCaT keratinocytes and RasV12/E1A-transfected primary keratinocytes. Cell proliferation increased after transfection with an HC Rac1 plasmid, which results in hyperactivated Rac1. The increase in proliferation was abolished by incubation with the Rac1 inhibitors NSC23766 or W56, or depletion of keratin 17 with shRNA. In contrast, proliferation of HaCaT cells decreased when Rac1 activity was inhibited by NSC23766 or W56. This decrease in proliferation was abolished by overexpression of keratin 17 (Figure 2E). These results suggested that Rac1 activity was crucial for keratinocyte cell proliferation, and in this process, keratin 17 was possibly a factor.

**Figure 3:**
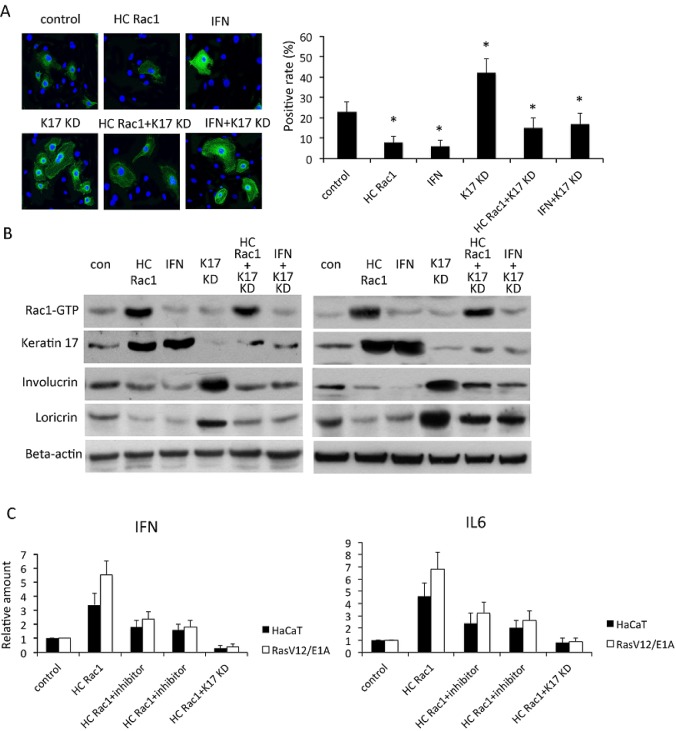
Rac1 is involved in keratinocyte differentiation A, In HaCaT cells stably expressing high-cycling (HC) Rac1 or small hairpin RNA against keratin 17 (KD), or treated with interferon (IFN), expression of involucrin (green) was investigated by immunofluorescence. DAPI (blue) counterstaining indicates nuclei. Scale bar: 5 mm. Positivity was calculated by counting positive cells out of all cells in each of five fields. * p<0.01 compare to control. B, Cells were transfected or treated as described in A and cytoplasmic proteins analyzed by western blot for keratin 17, involucrin, or loricrin. Rac1-GTP was evaluated by pull-down assay. C, HaCaT cells or RasV12/E1A-transfected keratinocytes were treated as described as in A and B. Production of IFN and IL6 was measured using real-time PCR and relative production determined by comparison with untreated cells.

Rac1 is important for differentiation of hair and skin stem cells [7, 18]. TPA treatment reduced expression of keratin1 and keratin10, as well as involucrin and loricrin, which are markers of epidermis differentiation, however, inhibition of Rac1 activity by NSC23766 induced keratin 1, keratin10, involucrin and loricrin throughout the epidermis even with TPA treatment (Figure 2B). In cultured keratinocytes, hyperactivation of Rac1, achieved by transfection with the HC Rac1 plasmid, or incubation of cells with interferon (IFN), which is a cytokine to stimulate inflammation, reduced expression of involucrin. These results indicated reduced differentiation of keratinocytes. Knockdown of keratin 17 significantly increased involucrin expression, suggesting enhanced keratinocyte differentiation, although this effect was inhibited by transfection with the HC Rac1 plasmid or incubation with IFN (Figure 3A). By western blot of HaCaT cells and RasV12/E1A-transfected keratinocytes, both HC Rac1 transfection and IFN treatment enhanced keratin 17 expression. However, knockdown of keratin 17 expression and incubation with IFN did not alter Rac1 activity. Both HC Rac1 transfection and IFN treatment reduced involucrin and loricrin expression. Depletion of keratin 17 enhanced involucrin and loricrin expression. These results suggested that keratin 17, regulated by Rac1 and IFN, is crucial for expression of involucrin and loricrin, which subsequently regulate differentiation in keratinocytes (Figure 3B).

IL6 and IFN are crucial for skin inflammation and other inflammation related cancer [19]. Sumida reported that reduction of IL6 receptor by mAb eliminates MDSCs in tumor-bearing mice. Therefore, we next asked whether Rac1 affected IL6 and IFN production. Production of IFN and IL6 increased after Rac1 was hyperactivated and production decreased after keratin 17 was depleted in HaCaT cells or RasV12/E1A-transfected keratinocytes (Figure 3C). These data suggested that Rac1 was important in the skin inflammatory reaction, possibly by regulation of keratin 17.

### Rac1 regulates Erk and Akt pathways in keratinocytes by regulation of keratin 17

**Figure 4:**
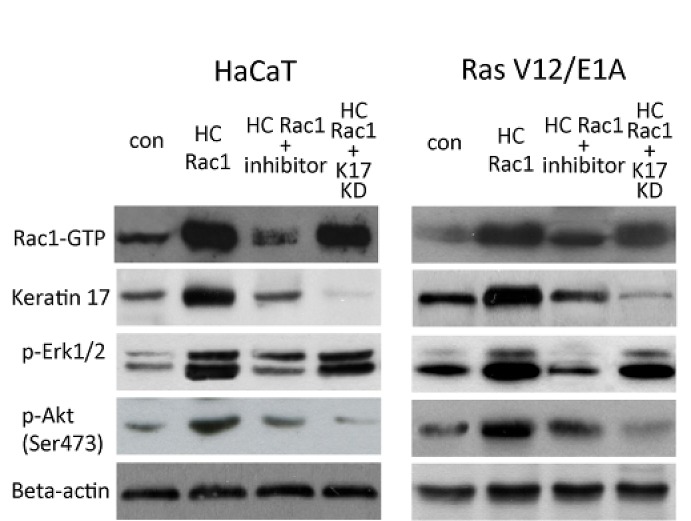
Rac1 and Erk and Akt signaling by keratin 17 HaCaT cells or RasV12/E1A-transfected keratinocytes were treated as described as in Figure 4 and proteins were analyzed by western blot for keratin 17, phosphorylated Erk1/2, or phosphorylated Akt (ser473). Rac1-GTP was evaluated using pull-down assay.

Erk1/2 and Akt activity are crucial for cell proliferation and differentiation [21]. We observed an increase in Erk1/2 and Akt phosphorylation corresponding to enhanced activity by using western blotting assay. This increase was partially reduced by knockdown of keratin 17. This result suggested that the Rac1 at least partly affected proliferation and differentiation *in vivo* and *in vitro*, possibly by regulation of keratin 17 (Figure 4).

### CD11b+Gr1+ cells might interact with Rac1 in keratinocytes

**Figure 5:**
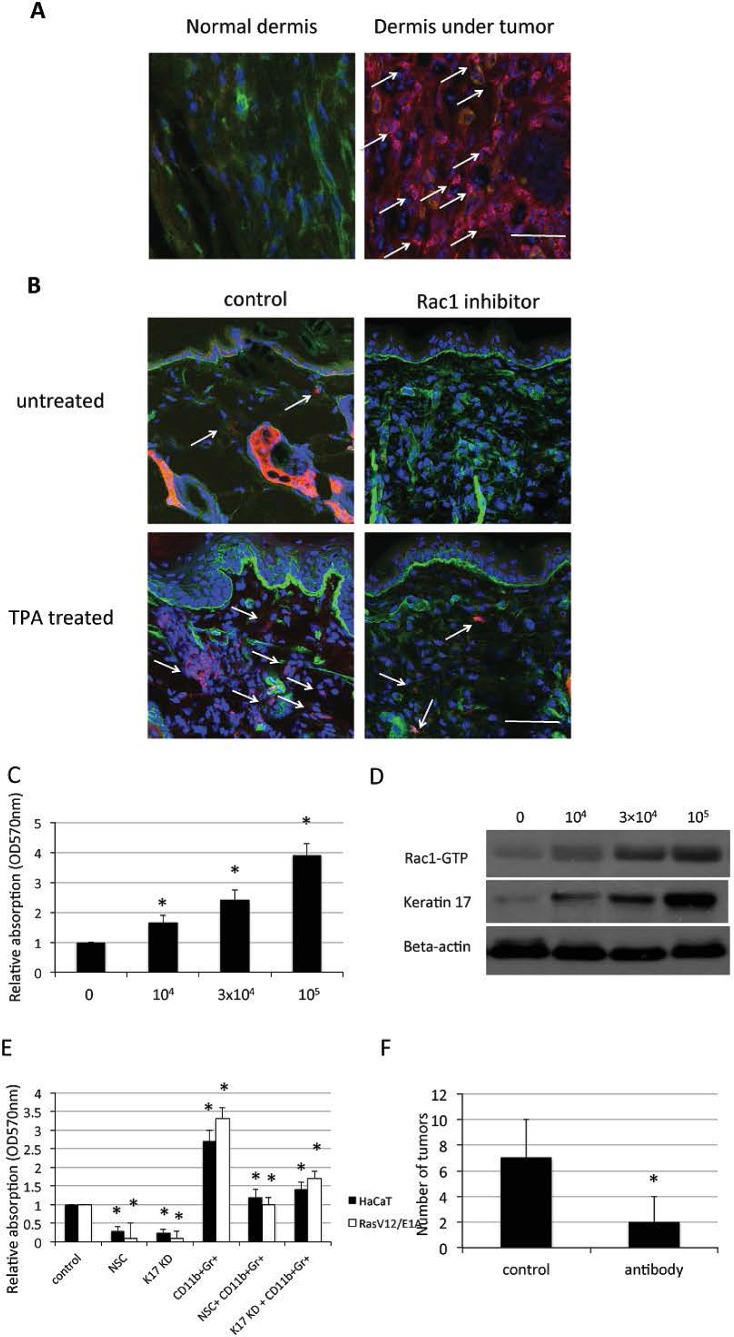
CD11b+Gr1+ cell infiltration and keratinocyte proliferation through Rac1 and keratin 17 A, Immunofluorescence for MPO positive MDSC cells (red) in the dermis of SCC patients. α6 (green), DAPI (blue) counterstaining indicates nuclei. Scale bar: 20 μm. B, 8 week old mice were treated with Rac inhibitor NSC23766 at 10mM 30min prior to TPA treatment or treated with TPA only, 48h later mice were sacrificed. Immunofluorescent staining was performed for Gr1+ cells (red) and α6 (green) in skin. DAPI (blue) counterstaining indicates nuclei. Scale bar: 20 μm. C, HaCaT cells were cocultured with the indicated number of CD11b+Gr1+ cells in 96-well plates for 72 h and proliferation of HaCaT cells was measured by crystal violet assay. Assays were performed in triplicate. The means with SD are shown (n=8 replicates/group). * p<0.01 compare to control. D, HaCaT cells were cocultured with the indicated number of CD11b+Gr1+ cells in 6-well plates for 24 h and proteins were analyzed by western blot for keratin 17. Rac1-GTP was evaluated using pull-down assay. E, HaCaT cells or RasV12/E1A-transfected keratinocytes were treated with Rac1 inhibitor NSC23766 (NSC), small hairpin RNA against keratin17 (KD), or cocultured with 10^4^ CD11b+Gr1+ cells in 96-well plates for 72 h with proliferation measured by crystal violet assay. F, Control (con) mice and mice treated by intraperitoneal injection with Gr1 monoclonal antibody every four days for 4 weeks. TPA was used treated mice for 2 weeks (n = 10). Shown is average number of tumors per mouse. * p<0.01 compare to control.

Given our results suggesting that Rac1 functioned in keratinocyte proliferation and differentiation, we asked whether Rac1 regulated an inflammatory microenvironment that promoted skin tumor formation. Myeloperoxidase (MPO) is a marker for human immature myeloid cells [11]. Immunohistochemistry staining for MPO in patient tumor and normal skin tissues showed that tumor tissues had more CD11b+Gr1+ cells infiltrating the dermis than normal skin (Figure 5A). In TPA-treated mice, significantly more CD11b+Gr1+ cells were found in the dermis than in untreated mice. Inhibition of Rac1 activity in mice skin reduced CD11b+Gr1+ cell accumulation in the dermis (Figure 5B).

To investigate the function of CD11b+Gr1+ cells in skin tumor formation, we incubated keratinocytes with different amounts of CD11b+Gr1+ cells, which isolated from tumors of mice, resulting in increased proliferation of keratinocytes (Figure 5C). Western blot results showed that enhancement of Rac1 activity and keratin 17 expression in keratinocytes depended on the number of CD11b+Gr1+ cells in the coincubation (Figure 5D). Inhibition of Rac1 activity and repression of keratin 17 expression partially blocked the increase in proliferation (Figure 5E).

We next investigated the function of CD11b+Gr1+ cells in the DMBA/TPA-induced mouse model. The results showed that ablation of CD11b+Gr1+ cells by intraperitoneal injection of a monoclonal antibody against Gr1 reduced skin tumor formation in mice (Figure 5F). These data indicated that CD11b+Gr1+ cells could be important for skin tumor formation through regulation of Rac1 activity.

### CD11b+Gr+1 cells activate Rac1 through regulation of Wnt signalling in keratinocytes

**Figure 6:**
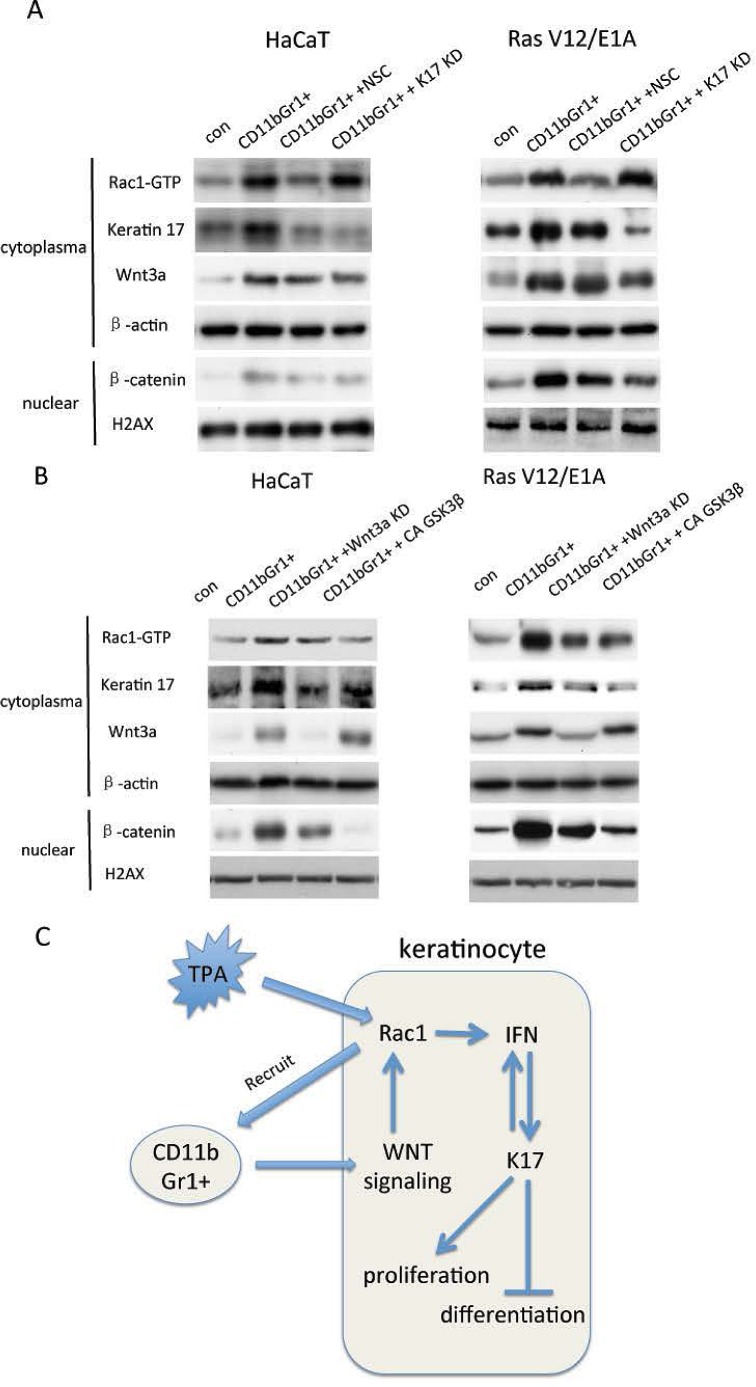
CD11b+Gr1+ cells, Rac1 activity and Wnt signaling in keratinocytes A, HaCaT cells or RasV12/E1A-transfected keratinocytes were treated with small hairpin RNA against keratin 17 (KD) or Rac1 inhibitor (NSC) and cocultured with 10^5^ CD11b+Gr1+ cells in 6-well plates for 24 h. Proteins were analyzed by western blot for keratin 17 and Wnt3a. Rac1-GTP was evaluated using pull-down assay. Nuclear proteins were extracted and subjected to western blot for β-catenin. B, HaCaT cells or RasV12/E1A-transfected keratinocytes were treated with small hairpin RNA against Wnt3a (KD) or constitutive active (CA) GSK3β and cocultured with 10^5^ CD11b+Gr1+ cells in 6-well plates for 24 h. Proteins were analyzed by western blot for keratin 17 and Wnt3a. Rac1-GTP was evaluated using pull-down assay. Nuclear proteins were extracted and subjected to western blot for β-catenin. C. Scheme of Rac1 and keratin17 function and relationship in skin carcinogenesis.

Wnt signaling has been demonstrated to be important for skin carcinogensis [12]. In keratinocytes, coculture with CD11b+Gr1+ cells induced overexpression of Wnt3a and β-catenin translocation into the nucleus. However, inhibition of Rac1 activity and repression of keratin 17 did not alter either Wnt3a expression and β-catenin translocation (Figure 6A). We knocked down Wnt3a expression and inhibited β-catenin translocation by constitutive activation of GSK3β in keratinocytes. Inhibition of Wnt signaling reduced Rac1 activity and keratin17 expression (Figure 6B). These results suggested that CD11b+Gr1+ cells might activate Rac1 activity and keratin17 expression in keratinocytes through regulation of the Wnt pathway.

## DISCUSSION

In this study, we have investigated the mechanism by which Rac1 promotes skin tumor formation. We found evidence suggesting that the Rac1 effect is mediated by enhancement of an IFN-keratin 17 loop, as well as recruitment of and interaction with CD11b+Gr1+ cells that induce inflammation and proliferation, but inhibit differentiation.

Hyperactivation of Rac1 in SCC tissues correlates with keratin 17 overexpression Treatment of DMBA/TPA results in tumor development and is accompanied by the induction of protumorigenic inflammation, which augments Wnt/β-catenin signaling [22]. Our findings suggested the mechanism of tumor promotion by Rac1 in this process. This model might be useful for other epithelial malignancies mediated by inflammation.

Rac1 is crucial for skin tumor formation possibly through regulation of an IFN-keratin 17 loop

Rac1 is overexpressed in many human tumors [17]. We previously showed that Rac1 is crucial for Ras-dependent skin tumor formation and regulates crosstalk between keratinocytes and immune cells [4, 17]. In this study, we examined the link between inflammation and skin tumor formation. We found that Rac1 activation but not expression is involved in skin tumor formation. Rac1 expression in skin tumor tissue was similar to that of in normal skin, but Rac1 activation in tumors was significantly higher than in normal skin. This result indicated that Rac1 activation rather than expression was associated with tumor formation. Furthermore, we correlated skin tumors with inflammatory stimulation. The DMBA/TPA-induced mouse skin tumor model is used to study multistage carcinogenesis and the functional interaction between inflammatory microenvironments and epithelial tumors. We found that when Rac1 activity was inhibited by NSC23766, fewer tumors were observed in the DMBA/TPA mouse model, suggesting involvement of Rac1 activity in tumor formation. In tumor tissues of patients, Rac1 activation is positively correlated with keratin 17 expression. *In vitro*, hyperactivation of Rac1 resulted in hyperproliferation and decreased differentiation of keratinocytes as well as enhanced production of IFN and IL6, which are crucial cytokines for skin inflammation. Thus, Rac1 might be a key molecular link between inflammation and skin tumor formation.

Rac1 functions in skin tumor formation through CD11b+Gr1+ cells

The role of CD11b+Gr1+ cells in tumor formation has been widely reported [23, 24]. We demonstrated that CD11b+Gr1+ cells might be recruited by hyperactivated Rac1 in keratinocytes. The dermis of patient tumor tissues showed significantly more CD11b+Gr1+ cells than normal tissues. In mice, TPA treatment enhanced Rac1 activity in the epidermis and increased the number of CD11b+Gr1+ cells in dermis. Treatment with a Rac1 inhibitor reduced the number of CD11b+Gr1+ cells, with or without TPA treatment. These data suggested that Rac1 activity was involved in recruitment and accumulation of CD11b+Gr1+ cells in the dermis.

To investigate the function of CD11b+Gr1+ cells in skin tumor formation *in vivo*, we treated mice with DMBA/TPA-induced skin tumors with antibody against CD11b+Gr1+ cells. Depletion of CD11b+Gr1+ cells resulted in significantly fewer tumors in the mice. *In vitro*, when HaCaT and RasV12/E1A-immortalized keratinocytes were incubated with CD11b+Gr1+ cells, the myeloid cells appeared to promote proliferation of keratinocytes, and increased numbers resulted in increased promotion. This effect on proliferation was abolished by inhibition of Rac1 or repression of keratin 17. These results indicated that keratinocyte proliferation might be driven, at least in part, by Rac1 and keratin 17, and promoted by CD11b+Gr1+ cells. Furthermore, activity of Rac1 in keratinocytes increased after coculture with CD11b+Gr1+ cells depending on the number of myeloid cells. This result suggested that Rac1 might recruit CD11b+Gr1+ cells, which in turn further activate Rac1 in keratinocytes. We postulate that this positive loop is indispensable to tumorigenesis in skin.

CD11b+Gr+11 cells activate Wnt signaling by regulation of Rac1 in keratinocytes.

Although evidence supports CD11b+Gr1+ cell activation of Rac1, the mechanism by which CD11b+Gr1+ cells regulate Rac1 activity is unclear. Piazza et al. (2012) reported that accumulation of CD11b+Gr1+ cells promotes tumor growth by increased provision of the Wnt ligand Wnt3a. We found that depletion of CD11b+Gr1+ cells resulted in reduced β-catenin staining in the nucleus of epidermal cells. Rac1 is reported to be essential for Wnt-driven expansion and transformation of intestinal stem cells [25] and Wnt5a promotes breast cancer cell migration via the Dvl2/Rab35/Rac1 signaling pathway [26]. In our study, inactivation of Rac1 or repression of keratin 17 did not alter Wnt3a expression or nuclear localization of β-catenin; on the other hand, repression of Wnt3a or inhibition of β-catenin nuclear translocation by activation of GSK3β reduced Rac1 activity. These results suggested that Rac1 in keratinocytes was activated by Wnt signaling which, at least in part, was activated by accumulation of CD11b+Gr1+ cells.

In conclusion, Rac1 appeared to promote skin tumor formation by activation of an IFN-keratin 17 loop and through recruitment of CD11b+Gr1+ cells. We propose that CD11b+Gr1+ cells activate Rac1 in keratinocytes by regulation of Wnt signalling.

## MATERIALS AND METHODS

### Tissue collection

Human SCC specimens were obtained from 156 patients in Xijing Hospital, Xi'an, China. Collection of patient samples was with informed written consent, according to an established protocol. All experimental procedures were approved by the Research Ethics Committee of Xijing Hospital.

### Animals

To establish a skin tumor model in mice, Crj: CD-1(ICR) mice (Biotechology & Cell Biology Institute, Shanghai, China)were treated with DMBA and TPA to induce skin tumors [17]. The backs of 8-week-old mice were shaved and 24 h later, 50 μg DMBA (Sigma, St Louis, MO, USA) in 200 μl acetone was applied. After a week, the backs of the mice were treated twice a week with 5 μg TPA (Sigma) in 200 μl acetone for 30 weeks. Untreated mice received acetone applications without DMBA or TPA. To inhibit Rac1 activity, the backs of the mice were treated with 10 mM NSC23766 (Tocris Bioscience, Ellisville, Missouri, USA) in acetone 30min prior to TPA treatment. Tumors were counted twice a week. Mice were sacrificed after 30 weeks. The tumor volume was calculated by the following formula: V =(L×W^2^)×0.5, where L is the length and W is the width of the tumor.

### Immunohistochemistry, BrdU and immunofluorescent staining

For immunohistochemistry and BrdU staining, paraffin sections of human tumor tissues and normal skin were stained according to standard protocols [17] with antibodies against keratin 17 (Cell Signaling). Streptavidin-conjugated secondary antibody was applied and visualization was with 3,3'-Diaminobenzidine (DAB). Images were analyzed with a Leica light microscope.

Immunofluoresence staining was performed using cryosections of mouse back skin at the end of DMBA/TPA treatment and cultured keratinocytes according to a previous protocol [17]. Primary antibodies were: keratin 17, keratin 6, keratin1, keratin 10 (Acris), involucrin, loricrin, Ly-6G (Gr-1), myeloperioxidase (MPO) (Abcam), CD45 and CD49f (integrin a6 chain) directly coupled to fluorescein isothiocyanate (BD Pharmingen, San Jose, CA, USA). Cy3-conjugated goat anti-rabbit IgG (Jackson Immuno- Research Laboratories, Inc., West Grove, PA, USA) was used as a secondary reagent. Nuclear DNA was visualized with DAPI (Sigma). Images were collected with a LSM700 confocal microscope (Zeiss, Oberkochen, Germany).

### Cell culturing and assays

Human keratinocyte HaCaT cells (ATCC) were cultured in DMEM supplemented with 10% heat-inactivated fetal bovine serum, 100 U/ml penicillin and 100 mg/ml streptomycin. Cells were incubated at 37°C in a humidified atmosphere (5% CO_2_). Primary keratinocytes were isolated from adult mice and cultured as described previously [17] and retrovirally transformed with H-Ras V12/E1A (vectors kindly provided by Professor Cord Brakebusch, University of Copenhagen), keratin 17-overexpressing plasmid (Gene Pharm Co. Ltd., China), or Tag5Amyc-GSK3b CA (Addgene) and selected with 1 mg/ml puromycin or G418 (Sigma) for 14 days. Treatment with Rac1 inhibitors NSC23766 at 50 μM or W56 (Upstate Biotechnology, Lake Placid, NY) 6 ng/ml was for 48 h. Transfections were carried out as described previously [27] using 5 μg/ml small hairpin (sh)RNA against keratin 17 with G418 (Sigma) selection at 200 μg/ml applied 24 h after transfection. EGFP-high-cycling (HC) Rac1 vector (a gift from Professor Cord Brakebusch, University of Copenhagen) was transfected and cells were sorted by flow cytometer. All experiments were independently repeated three times.

### Crystal violet assays

Cell survival was determined by crystal violet assay. Cells (5 x 10^3^ cells/well) were seeded in a 96-well plate for 72 h. Cells were fixed with 70% ethanol and incubated with medium containing 0.5% crystal violet in 20% methanol at room temperature. Excess dye was washed away and cell-bound crystal violet was extracted with 10% SDS. Absorbance was read using a microplate reader (Bio-Rad) at 570 nm.

### Western blot

Western blotting was according to standard protocols [28]. Tissue or cell proteins were incubated with antibodies against Rac1, keratin 17, involucrin, loricrin, p-Erk1/2(Thr202/Tyr204), or p-Akt(ser473) (Cell Signaling) overnight. Blots were washed, exposed to horseradish peroxidase-conjugated secondary antibodies (Santa Cruz Biotechnology) for 2 h, and examined by enhanced chemiluminescence (PerkinElmer). Bands were quantified using TotalLab TL100 software (Nonlinear Dynamics). Antibody against β-actin (Santa Cruz Biotechnology) was used to normalize protein amounts.

### Pull-down assay

Rac1 activity of epidermis lysates and cultured cells was determined as described previously [27]. GTP-bound, active Rac (Rac-GTP) was precipitated by binding to glutathione-coupled Sepharose beads. GTP-Rac1 was detected by immunoblotting with anti-Rac1 antibody.

### Real-time PCR

RNA was isolated from keratinocytes according to a standard protocol [4] using Geneelute mammalian Total RNA miniprep kits (Sigma). RNA was reverse transcribed by using a Revertaid H minus first strand cDNA synthesis kit K1632 (Fermentas). Real-time PCR was performed on the applied Biosystems 7300 Real Time PCR system using SYBR green incorporation. Ct values were calculated based on duplicates and normalized to the housekeeping gene *cycA.*

### Depletion of CD11b+Gr1+ cells and TPA treatment

To deplete Gr1+ cells, 6 mice were injected intraperitoneally with 10 mg/10 g body weight with anti-GR-1 mAb RB6-8C5 (BioXCell) or isotype control (IgG2b) every fourth day for 4 weeks. Then the backs of the mice were treated with 5 μg TPA (Sigma) in 200 μl acetone, or 10 mM NSC23766 (Tocris Bioscience, Ellisville, Missouri, USA) in acetone 30min prior to TPA treatment. TPA treatment was applied twice / week for 2 weeks.

### Isolation of CD11b+Gr1+ cells from skin tumors

Skin tumor samples from 6 tumors of mice were dissected and cut into small pieces after wash with PBS. Tissue fragments were digested in DMEM containing 200U/mL type I collagenase, 1% trypsin and 70U/mL DNase shaking at 37ºC for 2h. Then cells were filtered through a 70 μm cell strainer (BD Falcon) and washed twice in Flow Cytometry (FACS) staining medium (1x HBSS, 25 mM Hepes pH 7.2, 2% newborn bovine calf serum). Cells were analyzed directly by flow cytometry or stained with fluorescence-labeled antibodies specific for CD45 (EPFL Protein Core Facility) or Gr1 (EPFL Protein Core Facility) and sorted with a FACStarPlus flow cytometer (Becton Dickinson, Franklin Lakes, NJ).

### Statistical analysis

Data analysis was performed using a two-tailed unpaired *t*-test. Values were expressed as mean ± standard error of the mean (SEM). P < 0.01 was considered significant. Correlation between Rac1 activity and keratin17 was analyzed by using Spearman rank correlation coefficient method. All analysis were down by using SPSS13.0 software.

### Disclosure of Potential Conflicts of Interest

No potential conflicts of interest were disclosed.
